# Cefepime-Resistant *Pseudomonas aeruginosa*

**DOI:** 10.3201/eid1706.100358

**Published:** 2011-06

**Authors:** Ehimare Akhabue, Marie Synnestvedt, Mark G. Weiner, Warren B. Bilker, Ebbing Lautenbach

**Affiliations:** Author affiliation: University of Pennsylvania School of Medicine, Philadelphia, Pennsylvania, USA

**Keywords:** resistance, cefepime, Pseudomonas aeruginosa, risk factors, outcomes, bacteria, research

## MEDSCAPE CME

Medscape, LLC is pleased to provide online continuing medical education (CME) for this journal article, allowing clinicians the opportunity to earn CME credit.

This activity has been planned and implemented in accordance with the Essential Areas and policies of the Accreditation Council for Continuing Medical Education through the joint sponsorship of Medscape, LLC and Emerging Infectious Diseases. Medscape, LLC is accredited by the ACCME to provide continuing medical education for physicians.

Medscape, LLC designates this Journal-based CME activity for a maximum of 1 *AMA PRA Category 1 Credit(s)*^TM^. Physicians should claim only the credit commensurate with the extent of their participation in the activity.

All other clinicians completing this activity will be issued a certificate of participation. To participate in this journal CME activity: (1) review the learning objectives and author disclosures; (2) study the education content; (3) take the post-test and/or complete the evaluation at www.medscape.org/journal/eid; (4) view/print certificate.

**Release date: May 24, 2011; Expiration date: May 24, 2012**

## Learning Objectives

Upon completion of this activity, participants will be able to:

- Distinguish the prevalence of cefepime-resistant *P. aeruginosa*
- Analyze risk factors for the development of cefepime-resistant *P. aeruginosa*
- Evaluate the effects of cefepime-resistant *P. aeruginosa* on the risk for mortality.

## MEDSCAPE CME Editor

**P. Lynne Stockton, **Technical Writer/Editor, *Emerging Infectious Diseases. Disclosure: P. Lynne Stockton has disclosed no relevant financial relationships.*

## MEDSCAPE CME AUTHOR

**Charles P. Vega, MD**, Associate Professor; Residency Director, Department of Family Medicine, University of California, Irvine. *Disclosure: Charles P. Vega, MD, has disclosed no relevant financial relationships.*

## AUTHORS

*Disclosures:* ***Ehimare Akhabue, BA,*** *and* ***Marie Synnestvedt, PhD,*** *have disclosed no relevant financial relationships.* ***Mark G. Weiner, MD,*** *has disclosed the following relevant financial relationships: received grants for clinical research from Pfizer Inc.* ***Warren B. Bilker, PhD,*** *has disclosed the following relevant financial relationships: served as an advisor or consultant for Johnson & Johnson Pharmaceutical Research & Development, LLC, Hisamitsu Pharmaceuticals, and Avid Radiopharmaceuticals; received grants for clinical research from Novartis Pharmaceuticals Corporation, Takeda Pharmaceuticals North America, Inc., and Pfizer Inc.* ***Ebbing Lautenbach, MD, MPH,*** *has disclosed the following relevant financial relationships: received grants for clinical research from 3M Pharmaceuticals and AstraZeneca Pharmaceuticals LP.*
