# Supplementary material for: Cefepime-Resistant Pseudomonas aeruginosa
Source: Emerg Infect Dis. 2011 Jun;17(6):1037–43. doi: 10.3201/eid1706.100358 (PMC3320237; doi:10.3201/eid1706.100358)
Supplement: Related texts

# Research article: Cefepime-Resistant *Pseudomonas aeruginosa*

**DOI:** 10.3201/eid1706.100358

**Authors:** Ehimare Akhabue, Marie Synnestvedt, Mark G. Weiner, Warren B. Bilker, Ebbing Lautenbach

**Affiliations:** Author affiliation: University of Pennsylvania School of Medicine, Philadelphia, Pennsylvania, USA

## Abstract

Resistance to extended-spectrum cephalosporins complicates treatment of *Pseudomonas aeruginosa* infections. To elucidate risk factors for cefepime-resistant *P. aeruginosa* and determine its association with patient death, we conducted a case–control study in Philadelphia, Pennsylvania. Among 2,529 patients hospitalized during 2001–2006, a total of 213 (8.4%) had cefepime-resistant *P. aeruginosa* infection. Independent risk factors were prior use of an extended-spectrum cephalosphorin (p<0.001), prior use of an extended-spectrum penicillin (p = 0.005), prior use of a quinolone (p<0.001), and transfer from an outside facility (p = 0.01). Among those hospitalized at least 30 days, mortality rates were higher for those with cefepime-resistant than with cefepime-susceptible *P. aeruginosa* infection (20.2% vs. 13.2%, p = 0.007). Cefepime-resistant *P. aeruginosa* was an independent risk factor for death only for patients for whom it could be isolated from blood (p = 0.001). Strategies to counter its emergence should focus on optimizing use of antipseudomonal drugs.

^1^Current affiliation: Duke University School of Medicine, Durham, North Carolina, USA.

*Pseudomonas aeruginosa* is one of the most common gram-negative bacterial causes of health care–acquired infections (*1**–**3*). These infections result in high morbidity and mortality rates (*4**,**5*). When serious *P. aeruginosa* infections are suspected, early and appropriate antimicrobial drug therapy is crucial because inadequate drug selection has been associated with increased mortality rates (*6**,**7*). Complicating the empiric selection of adequate therapy is the increasing prevalence of antimicrobial drug resistance among *P. aeruginosa* (*8**–**10*). Even in initially susceptible strains, resistance can rapidly develop during treatment (*11**–**13*).

Cefepime, a fourth-generation cephalosphorin, is one of the few agents remaining that has reliable activity against *P. aeruginosa*. However, increased prevalence of resistance to cefepime among these organisms has been noted (*14**–**18*). As such, elucidating the epidemiology of cefepime-resistant *P. aeruginosa* is crucial to ensure that this agent remains a viable therapeutic option. Our goals were to identify risk factors for cefepime-resistant *P. aeruginosa* infections in the hospital setting and to describe the clinical effects of these infections.

## Methods

The study was performed at the Hospital of the University of Pennsylvania (HUP), a 725-bed tertiary-care center, and Penn Presbyterian Medical Center (PPMC), a 344-bed urban community hospital. Each hospital is located in Philadelphia, Pennsylvania, USA, and is part of the University of Pennsylvania Health System. The study was reviewed and approved by the University of Pennsylvania Institutional Review Board.

### Participants

To investigate risk factors for cefepime-resistant *P. aeruginosa*, we conducted a case–control study. We identified study participants through records obtained from the clinical microbiology laboratory at HUP, which performs bacterial cultures on all clinical specimens from HUP and PPMC. All adult patients with a positive *P. aeruginosa* culture result from January 1, 2001, through December 31, 2006, were eligible for inclusion. Each participant was included only one time; the first positive *P. aeruginosa* culture identified during the study period was used.

On the basis of our first study goal—identifying risk factors—we designated all participants with a cefepime-resistant *P. aeruginosa*–positive culture result as case-patients and all participants with a cefepime-susceptible *P. aeruginosa* culture result as controls. All eligible case-patients and controls were included according to the aforementioned eligibility criteria.

### Variables

To assess risk factor variables, we used a comprehensive clinical and administrative University of Pennsylvania health system database, which contains data for all hospitalizations since January 1, 2001, and has been used successfully for similar studies of antimicrobial drug resistance (*19**–**21*). Data elements obtained were age, sex, race, hospital (HUP or PPMC), admission as a transfer from another facility (i.e., outside hospital, long-term care facility, rehabilitation center), location within the hospital at the time of culture (i.e., intensive care unit or not intensive care unit), length of hospital stay before culture, prior admission to HUP or PPMC within the past 30 days, Charlson index (*22*), and all-patient refined–diagnosis-related group (APR-DRG) classification. The following concurrent conditions were also noted: renal insufficiency (serum creatinine level >2.0 mg/dL or requirement for dialysis), malignancy, diabetes, cirrhosis, congestive heart failure, chronic pulmonary disease, immunosuppressive therapy, and HIV infection. These variables were based on International Classification of Diseases, Ninth Revision codes; laboratory data; and pharmacy data.

### Drug Susceptibility Profiles

We documented antimicrobial drug susceptibility profiles, anatomic site of cultures, and any co-infections. Drug susceptibilities were conducted and interpreted by a semiautomated system (MicroScan WalkAway System, NC16 panel; Dade Behring, St. Louis, MO, USA) or disk-diffusion susceptibility testing in accordance with the criteria of the Clinical and Laboratory Standards Institute (*23*). Isolates with MIC = 16 (intermediate) or MIC >32 (resistant) were deemed resistant. A multidrug-resistant strain of *P. aeruginosa* was defined as a strain with resistance to >3 antimicrobial drug classes (*24*). We documented all antimicrobial drug treatment administered during the same inpatient admission for up to 30 days before the positive *P. aeruginosa* culture. We then categorized the drugs by individual agent, class, and spectrum of activity as follows: aminoglycosides (gentamicin, amikacin), quinolones (levofloxacin, ciprofloxacin), extended-spectrum penicillins (piperacillin-tazobactam), extended-spectrum cephalosporins (cefepime, ceftazidime), carbapenems (imipenem, meropenem), anaerobic therapy (amoxicillin/clavulanate, ampicillin/sulbactam, ceftriaxone, imipenem, meropenem, metronidazole, clindamycin), tetracyclines (doxycylcine), and macrolides (azithromycin, erythromycin) (*25*). During this study period, cefepime was the primary extended-spectrum cephalosporin used at HUP and PPMC, per formulary guidelines. For multivariable analyses, antimicrobial drugs were categorized by agent or class.

### Mortality Rates

To assess the relationship between cefepime-resistant *P. aeruginosa* and mortality rates, we performed a retrospective cohort study, designating the participants with cefepime-resistant *P. aeruginosa* as the exposed group and those with cefepime-susceptible *P. aeruginosa* as the unexposed group. We focused specifically on rates for those hospitalized at least 30 days.

### Statistical Analyses

We calculated the overall and annual prevalence of cefepime-resistant *P. aeruginosa* among all isolates identified during the study period. We then evaluated the annual prevalence of cefepime resistance over time by performing the χ^2^ test for trend (*26*).

To assess possible associations between potential risk factors and cefepime-resistant *P. aeruginosa*, we initially conducted bivariable analyses. Categorical variables were analyzed by using the Fisher exact test, and continuous variables were analyzed by using the Wilcoxon rank-sum test (*27*). The strength of each association was evaluated by calculating an odds ratio (OR) and a 95% confidence interval (CI). Multivariable analysis was performed by using forward stepwise multiple logistic regression (*28*). All variables with p<0.20 on bivariable analyses were considered for inclusion in the multivariable model. Backward stepwise multiple logistic regression was also performed to determine whether identification of risk factors varied with the approach to multivariable analysis. Because of the need to adjust for time at risk when investigating risk factors for antimicrobial drug resistance, we required the “duration of hospitalization prior to culture” variable to remain in the final model (*29*). We also analyzed the interaction between risk factor variables in the final model. Finally, to focus on those isolates likely to represent clinical infection, as per Centers for Disease Control and Prevention criteria, we repeated the analyses on blood isolates only (*30*).

To assess the association between cefepime-resistant *P. aeruginosa* and mortality rates for those hospitalized at least 30 days, we conducted bivariable and multivariable analyses in a similar fashion as for the case–control study. As we did for the case–control study, we repeated the analyses on blood isolates only.

We considered a 2-tailed p<0.05 significant. We used STATA version 10.0 (StataCorp, College Station, TX, USA) to perform the statistical analysis.

## Results

During the study period, culture results were positive for *P. aeruginosa* , and cefepime susceptibility was tested for 2,529 patients. Median patient age was 61 years (95% CI 60–62), and 1,439 (56.9%) patients were male. Regarding race and/or ethnicity, 1,116 (44.4%) were white, 848 (33.7%) were African American, 30 (1.2%) were Asian, 29 (1.2%) were Hispanic, and the rest were identified as other or unknown. Among all participants, 1,984 (78.5%) were hospitalized at HUP and 545 (21.6%) were hospitalized at PPMC.

*P. aeruginosa* isolates came from the following anatomic sites: respiratory tract (247 [35.5%]), urine (763 [30.2%]), wound (467 [18.5%]), blood (248 [9.8%]), tissue (120 [4.7%]), and other (35 [1.3%]). Among the 2,529 isolates, 213 (8.4%) exhibited cefepime resistance and 339 (13.4%) exhibited multidrug resistance. Annual prevalence of *P. aeruginosa* cefepime resistance over time showed no significant trend (p = 0.99; Figure).

**Figure:** Prevalence of infection with cefepime-resistant *Pseudomonas aeruginosa*, Philadelphia, PA, USA, 2001–2006. p = 0.9946 for trend.

Using bivariate analysis to compare exposures, we found several differences between cefepime-resistant and cefepime-susceptible *P. aeruginosa* (Table 1). Specifically, participants with cefepime-resistant *P. aeruginosa* were more likely to have received an extended-spectrum cephalosporin, extended-spectrum penicillin, or quinolone. Multivariate analysis indicated that prior use of an extended-spectrum cephalosphorin had the strongest association with cefepime-resistant *P. aeruginosa* (adjusted OR 2.18, 95% CI 1.57–3.04; p<0.001) (Table 2). Independently associated with cefepime-resistant *P. aeruginosa* were prior use of an extended-spectrum penicillin or a quinolone and transfer from an outside facility (Table 2). No substantive differences were found in the final model when analyses were limited to blood isolates.

**Table 1:** Bivariable analysis comparing patient exposures to cefepime-resistant and cefepime-susceptible *Pseudomonas aeruginosa,* Philadelphia, PA, USA, 2001–2006*

Variable	No. (%) case-patients, n = 213	No. (%) controls, n = 2,316	OR (95% CI)	p value†
General				
Male sex	132 (62.0)	1,307 (56.4)	1.26 (0.94–1.70)	0.13
Race, white	95/207 (45.9)	1021/2,270 (45.0)	1.04 (0.77–1.39)	0.83
Hospital, PPMC	44 (20.7)	501 (21.6)	0.94 (0.65–1.34)	0.79
Transfer from another facility‡	73/212 (34.4)	509/2,304 (22.1)	1.85 (1.35–2.52)	<0.001
In ICU at time of culture	104/202 (51.5)	831/2,132 (39.0)	1.66 (1.23–2.24)	0.001
Prior hospitalization in past 30 d	60 (28.2)	526 (22.7)	1.33 (0.96–1.84)	0.07
APR-DRG§	152 (71.4)	1,328 (57.5)	1.84 (1.34–2.55)	<0.001
Concurrent illness				
Renal insufficiency	34 (16.0)	305 (13.2)	1.25 (0.82–1.86)	0.25
Malignancy	22 (10.3)	358 (15.5)	0.63 (0.38–0.99)	0.05
Diabetes	43 (20.2)	511 (22.1)	0.89 (0.62–1.28)	0.60
Liver disease	9 (4.2)	46 (2.0)	2.18 (0.92–4.58)	0.04
Congestive heart failure	2 (0.9)	37 (1.6)	0.58 (0.07–2.29)	0.77
Chronic pulmonary disease	52 (24.4)	453 (19.6)	1.33 (0.94–1.86)	0.11
Immunosuppressive therapy	39 (18.3)	256 (11.1)	1.80 (1.21–2.63)	0.004
HIV infection	5 (2.4)	54 (2.3)	1.01 (0.31–2.54)	>0.99
Antimicrobial drug use¶				
Any	150 (70.4)	1,458 (63.0)	1.40 (1.02–1.93)	0.03
Aminoglycoside	38 (17.8)	382 (16.5)	1.10 (0.74–1.60)	0.63
Quinolones	58 (27.2)	290 (12.5)	2.61 (1.85–3.65)	<0.001
Extended-spectrum penicillins	31 (14.6)	125 (5.4)	2.99 (1.89–4.60)	<0.001
Extended-spectrum cephalosporin	80 (37.6)	401 (17.3)	2.87 (2.10–3.90)	<0.001
Prior carbapenem	14 (6.6)	58 (2.5)	2.74 (1.38–5.08)	0.002
Prior anaerobic therapy	111 (52.1)	896 (38.7)	1.72 (1.29–2.31)	<0.001
Prior tetracyclines	1 (0.5)	18 (0.8)	0.60 (0.01–3.85)	>0.99
Prior macrolide	8 (3.8)	117 (5.1)	0.73 (0.31–1.52)	0.51

*OR, odds ratio; CI, confidence interval; PPMC, Penn Presbyterian Medical Center; ICU, intensive care unit; APR-DRG: all-patient refined-diagnosis–related group. Case-patients, those with cefepime-resistant *P. aeruginosa;* median (interquartile range) duration of stay before culture 8 (4–12) d; and Charlson index 2. Controls, those with cefepime-susceptible *P. aeruginosa* (interquartile range) duration of stay before culture 4 (4–5) d; and Charlson index 2. †Fisher exact test for categorical variables; Wilcoxon rank-sum test for continuous variables. ‡Outside hospital, long-term care facility, or rehabilitation center. §Patients in the extreme illness category. ¶Inpatient use within previous 30 d before culture during same hospitalization.

**Table 2:** Multivariable model of risk factors for cefepime-resistant *Pseudomonas aeruginosa* infection*,* Philadelphia, PA, USA, 2001–2006*

Variable	Unadjusted OR	Adjusted OR (95% CI)	p value
Prior use of extended-spectrum cephalosporin	2.87	2.18 (1.57–3.04)	<0.001
Prior use of extended-spectrum penicillin	2.99	1.91 (1.22–2.99)	0.005
Prior use of quinolone	2.61	1.96 (1.38–2.78)	<0.001
Prior use of carbapenem	2.74	1.70 (0.90–3.21)	0.10
Transfer from outside facility	1.85	1.49 (1.09–2.04)	0.01
Length of hospital stay before culture	NA	1.00 (0.99–1.01)†	0.81

*OR, odds ratio; CI, confidence interval; NA, not applicable. No substantive changes were found when above analyses were limited to bloodstream isolates only. †Odds associated with each 1-day increase in hospital stay.

The overall mortality rate among participants was 13.8% (348/2,529). The mortality rate for participants with cefepime-resistant *P. aeruginosa* was 20.2% (43/213) and for participants with cefepime-susceptible *P. aeruginosa* was 13.2% (305/2,316) (relative risk [RR] 1.53, 95% CI 1.15– 2.04; p = 0.007). After controlling for significant confounders in the multivariate analysis, cefepime-resistant *P. aeruginosa* was no longer associated with death (Table 3). However, the association between cefepime-resistant *P. aeruginosa* and death varied significantly, depending on whether the isolate was from the blood or elsewhere. When analyses were restricted to blood isolates, a significant independent association between cefepime-resistant *P. aeruginosa* and death was found (RR 15.55, 95% CI 3.10–77.89; p = 0.001] (Table 4).

**Table 3:** Multivariable model of association between infection with cefepime-resistant *Pseudomonas aeruginosa* and death, Philadelphia, PA, USA, 2001–2006*

Variable	Adjusted OR (95% CI)	p value
Cefepime-resistant organism	1.28 (0.86–1.90)	0.232
Patient in ICU at time of culture	2.33 (1.75–3.10)	<0.001
APR-DRG	11.29 (6.53–19.50)	<0.001
Patient transfer from outside hospital	1.38 (1.05–1.81)	0.021
Length of hospital stay before culture	0.99 (0.98–1.00)	0.231

*OR, odds ratio; CI, confidence interval; ICU, intensive care unit; APR-DRG, all-patient refined-diagnosis–related group.

**Table 4:** Multivariable model of cefepime-resistant *Pseudomonas aeruginosa* infection and death (blood isolates only), Philadelphia, PA, USA, 2001–2006*

Variable	Adjusted OR (95% CI)	p value
Cefepimeiresistant organism	15.55 (3.10–77.89)	0.001
Patient in ICU at time of culture	3.22 (1.50–6.91)	0.003
APR-DRG	4.48 (1.60–12.60)	0.004
Patient transfer from outside hospital	1.26 (0.56–2.86)	0.57
Length of hospital stay before culture	1.01 (0.99–1.04)	0.80

*OR, odds ratio; CI, confidence interval; ICU, intensive care unit; APR-DRG, all-patient refined-diagnosis–related group.

## Discussion

We found the following to be significant factors independently associated with isolation of a cefepime-resistant *P. aeruginosa* strain in culture of a clinical sample in the hospital setting: prior use of extended-spectrum cephalosphorins, extended-spectrum penicillins, or fluoroquinolones; and transfer from an outside facility. We also demonstrated that cefepime-resistant *P. aeruginosa* was independently associated with increased deaths among patients hospitalized for >30 days but only for those for whom *P. aeruginosa* was isolated from blood.

Past studies have found an association between use of an antipseudomonal agent and emergence of resistance to that same agent (*19**,**31**,**32*). Past studies have also demonstrated that *P. aeruginosa* resistance to 1 class of antimicrobial drugs is often associated with resistance to other classes (*11**,**33*). The tendency for health care–acquired *P. aeruginosa* to become resistant to drugs from multiple classes is well known, and several molecular mechanisms for its intrinsic and acquired resistance have been suggested (*10*). Our findings not only suggest that prior treatment with cefepime in itself is associated with subsequent emergence of cefepime-resistant *P. aeruginosa* but that even prior exposure to certain antipseudomonal agents in other classes is associated. Our results emphasize that to devise strategies that prevent further emergence of cefepime-resistant *P. aeruginosa*, recent prior use of antipseudomonal agents within the same class and from certain other classes must be recognized. The effect of curtailing use of non–β-lactam agents (i.e., fluoroquinolones) on cefepime-resistant *P. aeruginosa* prevalence should be formally assessed.

We also found that transfer from an outside facility was associated with cefepime-resistant *P. aeruginosa* infection. Because transferred patients potentially came from another hospital or from a long-term care facility, these patients might have been more likely to already be colonized with cefepime-resistant *P. aeruginosa* at the time of admission. That antimicrobial drug resistance is common in long-term care facilities is well known (*34*). Further work focusing specifically on antimicrobial drug–resistant *P. aeruginosa* infections in long-term care settings is warranted.

The significant association between cefepime-resistant *P. aeruginosa* infection and increased mortality rates (limited to those patients with *P. aeruginosa* bacteremia) may result from the fact that bacteremia is a more serious infection than, for example, a urinary tract infection. Alternatively, these results might be explained by noting that a blood isolate is more likely to represent a true infection than is an isolate from other anatomic sites, where isolates are more likely to represent colonization. Nonetheless, our results emphasize the potential serious effect of cefepime-resistant *P. aeruginosa* infection and the need for strategies to combat its further emergence.

This study has several potential limitations. The first is the ongoing, and appropriate, debate regarding the selection of the control group for case–control studies investigating the association between prior antimicrobial drug use and resistance. Like Harris and et al., we believe that selection of the control group depends on the study question (*29**,**35*). In our study, the main question was “What are the risk factors for cefepime resistance among all clinical isolates of *P. aeruginosa* in the hospital setting?” Thus, we selected patients with cefepime-susceptible *P. aeruginosa* infection as controls.

Another potential limitation is selection bias, which is always a concern in case–control studies. We believe that any such bias was minimized by the fact that every patient with a *P. aeruginosa* isolate was eligible for inclusion. Furthermore, all isolates were identified in the clinical microbiology laboratory at HUP, which processes all inpatient cultures at the participating study sites.

Misclassification bias is also a concern in case–control studies. However, the categorization of case-patients and controls and their exposure status was based entirely on preexisting clinical data from the clinical microbiology laboratory. The antimicrobial drug–susceptibility profiles were determined before study initiation, so determination of case and control status did not influence these profiles. Furthermore, case-patients and controls were selected without knowledge of their status regarding risk factors of interest. Thus, we believe any differential misclassification bias was unlikely.

Identification of participants in this study was based solely on clinical cultures. As such, that all of these cultures represented true infection is unlikely. For this reason, we performed additional analyses, focusing only on *P. aeruginosa* blood isolates because these would be expected to meet Centers for Disease Control and Prevention criteria for infection. The results of these secondary analyses did not differ substantively from the primary analyses investigating risk factors for cefepime-resistant *P. aeruginosa*. Finally, all patients in this study were admitted to either HUP or PPMC. Thus, our findings can only be generalized to similar academic centers. One must also keep in mind the differing resistance profiles at any given institution.

In conclusion, cefepime-resistant *P. aeruginosa* will negatively affect clinical outcomes, and strategies to counter its emergence are needed. Recognizing recent prior use of antipseudomonal agents, both within the same class and from certain other classes, is needed for devising successful interventions.

## References

[1] Gaynes R, Edwards JR. Overview of nosocomial infections caused by gram-negative bacilli. Clin Infect Dis. 2005;41:848–54. 10.1086/43280316107985

[2] Fluit AC, Jones ME, Schmitz FJ, Acar J, Gupta R, Verhoef J. Antimicrobial susceptibility and frequency of occurrence of clinical blood isolates in Europe from the SENTRY antimicrobial surveillance program, 1997 and 1998. Clin Infect Dis. 2000;30:454–60. 10.1086/31371010722427

[3] Streit JM, Jones RN, Sader HS, Fritsche TR. Assessment of pathogen occurrences and resistance profiles among infected patients in the intensive care unit: report from the SENTRY Antimicrobial Surveillance Program (North America, 2001). Int J Antimicrob Agents. 2004;24:111–8. 10.1016/j.ijantimicag.2003.12.01915288308

[4] Crouch Brewer S, Wunderink RG, Jones CB, Leeper KV Jr. Ventilator-associated pneumonia due to *Pseudomonas aeruginosa.* Chest. 1996;109:1019–29. 10.1378/chest.109.4.10198635325

[5] Osmon S, Ward S, Fraser VJ, Kollef MH. Hospital mortality for patients with bacteremia due to *Staphylococcus aureus* or *Pseudomonas aeruginosa.* Chest. 2004;125:607–16. 10.1378/chest.125.2.60714769745

[6] Micek ST, Lloyd AE, Ritchie DJ, Reichley RM, Fraser VJ, Kollef MH. *Pseudomonas aeruginosa* bloodstream infection: importance of appropriate initial antimicrobial treatment. Antimicrob Agents Chemother. 2005;49:1306–11. 10.1128/AAC.49.4.1306-1311.200515793102PMC1068618

[7] Vidal F, Mensa J, Almela M, Martínez JA, Marco F, Casals C, Epidemiology and outcome of *Pseudomonas aeruginosa* bacteremia, with special emphasis on the influence of antibiotic treatment. Analysis of 189 episodes. Arch Intern Med. 1996;156:2121–6. 10.1001/archinte.156.18.21218862105

[8] National Nosocomial Infections Surveillance System. National Nosocomial Infections Surveillance (NNIS) System report, data summary from January 1992 through June 2004, issued October 2004. Am J Infect Control. 2004;32:470–85. 10.1016/j.ajic.2004.10.00115573054

[9] Karlowsky JA, Jones ME, Thornsberry C, Evangelista AT, Yee YC, Sahm DF. Stable antimicrobial susceptibility rates for clinical isolates of *Pseudomonas aeruginosa* from the 2001–2003 Tracking Resistance in the United States Today surveillance studies. Clin Infect Dis. 2005;40(Suppl 2):S89–98. 10.1086/42618815712102

[10] McGowan JE, Jr. Resistance in nonfermenting gram-negative bacteria: multidrug resistance to the maximum. Am J Med. 2006;119(6 Suppl 1):S29–36; discussion S62–70.10.1016/j.amjmed.2006.03.01416735148

[11] Trouillet JL, Vuagnat A, Combes A, Kassis N, Chastre J, Gibert C. *Pseudomonas aeruginosa* ventilator-associated pneumonia: comparison of episodes due to piperacillin-resistant versus piperacillin-susceptible organisms. Clin Infect Dis. 2002;34:1047–54. 10.1086/33948811914992

[12] Carmeli Y, Troillet N, Eliopoulos GM, Samore MH. Emergence of antibiotic-resistant *Pseudomonas aeruginosa*: comparison of risks associated with different antipseudomonal agents. Antimicrob Agents Chemother. 1999;43:1379–82.1034875610.1128/aac.43.6.1379PMC89282

[13] Fink MP, Snydman DR, Niederman MS, Leeper KV Jr, Johnson RH, Heard SO, Treatment of severe pneumonia in hospitalized patients: results of a multicenter, randomized, double-blind trial comparing intravenous ciprofloxacin with imipenem-cilastatin. The Severe Pneumonia Study Group. Antimicrob Agents Chemother. 1994;38:547–57.820385310.1128/aac.38.3.547PMC284496

[14] American Thoracic Society; Infectious Diseases Society of America. Guidelines for the management of adults with hospital-acquired, ventilator-associated, and healthcare-associated pneumonia. Am J Respir Crit Care Med. 2005;171:388–416. 10.1164/rccm.200405-644ST15699079

[15] Neuhauser MM, Weinstein RA, Rydman R, Danziger LH, Karam G, Quinn JP. Antibiotic resistance among gram-negative bacilli in US intensive care units: implications for fluoroquinolone use. JAMA. 2003;289:885–8. 10.1001/jama.289.7.88512588273

[16] Ramphal R, Hoban DJ, Pfaller MA, Jones RN. Comparison of the activity of two broad-spectrum cephalosporins tested against 2,299 strains of *Pseudomonas aeruginosa* isolated at 38 North American medical centers participating in the SENTRY Antimicrobial Surveillance Program, 1997–1998. Diagn Microbiol Infect Dis. 2000;36:125–9. 10.1016/S0732-8893(99)00118-210705055

[17] Roberts JA, Webb SA, Lipman J. Cefepime versus ceftazidime: considerations for empirical use in critically ill patients. Int J Antimicrob Agents. 2007;29:117–28. 10.1016/j.ijantimicag.2006.08.03117158033

[18] Sader HS, Fritsche TR, Jones RN. Potency and spectrum trends for cefepime tested against 65,746 clinical bacterial isolates collected in North American medical centers: results from the SENTRY Antimicrobial Surveillance Program (1998–2003). Diagn Microbiol Infect Dis. 2005;52:265–73. 10.1016/j.diagmicrobio.2005.02.00316105569

[19] Gasink LB, Fishman NO, Weiner MG, Nachamkin I, Bilker WB, Lautenbach E. Fluoroquinolone-resistant *Pseudomonas aeruginosa*: assessment of risk factors and clinical impact. Am J Med. 2006;119:526 e19–25.10.1016/j.amjmed.2005.11.02916750968

[20] Lautenbach E, Synnestvedt M, Weiner MG, Bilker WB, Vo L, Schein J, Imipenem resistance in *Pseudomonas aeruginosa*: emergence, epidemiology, and impact on clinical and economic outcomes. Infect Control Hosp Epidemiol. 2010;31:47–53. 10.1086/64902119951202

[21] Lautenbach E, Weiner MG, Nachamkin I, Bilker WB, Sheridan A, Fishman NO. Imipenem resistance among *Pseudomonas aeruginosa* isolates: risk factors for infection and impact of resistance on clinical and economic outcomes. Infect Control Hosp Epidemiol. 2006;27:893–900. 10.1086/50727416941312

[22] Deyo RA, Cherkin DC, Ciol MA. Adapting a clinical comorbidity index for use with ICD-9-CM administrative databases. J Clin Epidemiol. 1992;45:613–9. 10.1016/0895-4356(92)90133-81607900

[23] National Committee for Clinical Laboratory Standards (NCCLS). Performance standards for antimicrobial susceptibility testing. NCCLS approved standard M100–S11. Wayne (PA): The Committee; 2001.

[24] Falagas ME, Koletsi PK, Bliziotis IA. The diversity of definitions of multidrug-resistant (MDR) and pandrug-resistant (PDR) *Acinetobacter baumannii* and *Pseudomonas aeruginosa.* J Med Microbiol. 2006;55:1619–29. 10.1099/jmm.0.46747-017108263

[25] MacAdam H, Zaoutis TE, Gasink LB, Bilker WB, Lautenbach E. Investigating the association between antibiotic use and antibiotic resistance: impact of different methods of categorising prior antibiotic use. Int J Antimicrob Agents. 2006;28:325–32. 10.1016/j.ijantimicag.2006.04.01416934439

[26] Armitage P. Test for linear trend in proportions and frequencies. Biometrics. 1955;11:375–86. 10.2307/3001775

[27] Kleinbaum DK, Kupper LL, Morgenstern H. Epidemiologic research: principles and quantitative methods. New York: Van Nostrand Reinhold; 1982.

[28] Hosmer DLS. Applied logistic regression. New York: Wiley and Sons; 1989.

[29] Harris AD, Karchmer TB, Carmeli Y, Samore MH. Methodological principles of case–control studies that analyzed risk factors for antibiotic resistance: a systematic review. Clin Infect Dis. 2001;32:1055–61. 10.1086/31960011264034

[30] Garner JS, Jarvis WR, Emori TG, Horan TC, Hughes JM. CDC definitions for nosocomial infections, 1988. Am J Infect Control. 1988;16:128–40. 10.1016/0196-6553(88)90053-32841893

[31] El Amari EB, Chamot E, Auckenthaler R, Pechère JC, Van Delden C. Influence of previous exposure to antibiotic therapy on the susceptibility pattern of *Pseudomonas aeruginosa* bacteremic isolates. Clin Infect Dis. 2001;33:1859–64. 10.1086/32434611692297

[32] Harris AD, Smith D, Johnson JA, Bradham DD, Roghmann MC. Risk factors for imipenem-resistant *Pseudomonas aeruginosa* among hospitalized patients. Clin Infect Dis. 2002;34:340–5. 10.1086/33823711774081

[33] López-Dupla M, Martínez JA, Vidal F, Almela M, Soriano A, Marco F, Previous ciprofloxacin exposure is associated with resistance to β-lactam antibiotics in subsequent *Pseudomonas aeruginosa* bacteremic isolates. Am J Infect Control. 2009;37:753–8. 10.1016/j.ajic.2009.02.00319487049

[34] Strausbaugh LJ, Crossley KB, Nurse BA, Thrupp LD. Antimicrobial resistance in long-term-care facilities. Infect Control Hosp Epidemiol. 1996;17:129–40. 10.1086/6472578835450

[35] Harris AD, Samore MH, Lipsitch M, Kaye KS, Perencevich E, Carmeli Y. Control-group selection importance in studies of antimicrobial resistance: examples applied to *Pseudomonas aeruginosa*, enterococci, and *Escherichia coli.* Clin Infect Dis. 2002;34:1558–63. 10.1086/34053312032889

# Research article: Cefepime-Resistant *Pseudomonas aeruginosa*

**DOI:** 10.3201/eid1706.100358

**Authors:** Ehimare Akhabue, Marie Synnestvedt, Mark G. Weiner, Warren B. Bilker, Ebbing Lautenbach

**Affiliations:** Author affiliation: University of Pennsylvania School of Medicine, Philadelphia, Pennsylvania, USA

## Earning CME Credit

To obtain credit, you should first read the journal article. After reading the article, you should be able to answer the following, related, multiple-choice questions. To complete the questions and earn continuing medical education (CME) credit, please go to www.medscape.org/journal/eid. Credit cannot be obtained for tests completed on paper, although you may use the worksheet below to keep a record of your answers. You must be a registered user on Medscape.org. If you are not registered on Medscape.org, please click on the New Users: Free Registration link on the left hand side of the website to register. Only one answer is correct for each question. Once you successfully answer all post-test questions you will be able to view and/or print your certificate. For questions regarding the content of this activity, contact the accredited provider, CME@medscape.net. For technical assistance, contact CME@webmd.net. American Medical Association’s Physician’s Recognition Award (AMA PRA) credits are accepted in the US as evidence of participation in CME activities. For further information on this award, please refer to http://www.ama-assn.org/ama/pub/category/2922.html. The AMA has determined that physicians not licensed in the US who participate in this CME activity are eligible for *AMA PRA Category 1 Credits*™. Through agreements that the AMA has made with agencies in some countries, AMA PRA credit is acceptable as evidence of participation in CME activities. If you are not licensed in the US and want to obtain an AMA PRA CME credit, please complete the questions online, print the certificate and present it to your national medical association.

## Article Title: Cefepime-Resistant *Pseudomonas aeruginosa*

## CME Questions

**1. You are seeing a 61-year-old man admitted for pneumonia. His blood culture is now growing *Pseudomonas aeruginosa*, and you are concerned regarding the possibility of antimicrobial resistance of this organism.What was the approximate rate of resistance to cefepime among isolates of *P. aeruginosa* in the current study?**A. Less than 1%B. 8%C. 22%D. 47%**2. Which of the following variables independently increased the risk for *P. aeruginosa* resistance to cefepime in the current study?**A. Male sexB. Diagnosis of pneumoniaC. Higher Charlson index scoreD. Transfer from another facility**3. The patient was treated with antibiotics as an outpatient prior to hospital admission. Prior treatment with which classes of antibiotics was found to increase the risk for cefepime-resistant *P. aeruginosa* (CRPA) in the current study?**A. Aminoglycosides onlyB. Extended-spectrum cephalosporins onlyC. Extended-spectrum cephalosporins, extended-spectrum penicillins, and quinolonesD. Aminoglycosides, extended-spectrum penicillins, and macrolides**4. The patient is diagnosed with CRPA. What does the current study suggest regarding the effect of CRPA vs. cefepime-sensitive *P. aeruginosa* on the risk for mortality?**A. CRPA did not confer a higher risk for mortality in any analysisB. Only older patients with CRPA were at a higher risk for deathC. Only patients with blood isolates for CRPA were at a higher risk for deathD. Any infection with CRPA was associated with a higher risk for death

## Activity Evaluation

**Table:** 

1. The activity supported the learning objectives.				
Strongly Disagree				Strongly Agree
1	2	3	4	5
2. The material was organized clearly for learning to occur.				
Strongly Disagree				Strongly Agree
1	2	3	4	5
3. The content learned from this activity will impact my practice.				
Strongly Disagree				Strongly Agree
1	2	3	4	5
4. The activity was presented objectively and free of commercial bias.				
Strongly Disagree				Strongly Agree
1	2	3	4	5
